# Uncovering a hidden danger: a case report of diffuse coronary spasm concealing spontaneous coronary artery dissection

**DOI:** 10.1186/s43044-024-00514-1

**Published:** 2024-07-04

**Authors:** Paolo Desario, David Rutigliano, Vincenzo Palumbo, Pasquale Caldarola

**Affiliations:** Cardiology Department, San Paolo Hospital, Via Caposcardicchio sn, 70132 Bari, Italy

**Keywords:** SCAD, Coronary artery spasm, CCTA, OCT, IVUS, Case report

## Abstract

**Background:**

Over recent years, spontaneous coronary artery dissection (SCAD) has emerged as a no longer rare cause of acute coronary syndrome (ACS). On the other hand, coronary artery spasm (CAS) is the main cause of ischemic heart disease with non-obstructive coronary lesions. Clinical manifestations of both vary from stable angina to ACS or, rarely, sudden cardiac death. These entities may be underdiagnosed on a coronary angiography.

**Case presentation:**

We report the case of a young woman presenting with acute chest pain and no coronary risk factors. Angiography revealed a focal subcritical stenosis of the right coronary artery. Coronary wiring resulted in diffuse and critical spasm. However, optical coherence tomography (OCT) and intravascular ultrasound (IVUS) showed extensive SCAD. She was therefore treated conservatively. On the fourth day, cardiac computed tomography angiography **(**CCTA) excluded disease progression, and then she was discharged on medical therapy.

**Conclusions:**

Combined IVI plays a vital role in providing accurate and detailed visualization of the coronary anatomy and thus allowing for more precise diagnosis, risk stratification, and treatment planning. CCTA can be considered a valuable tool in the noninvasive follow-up of SCAD.

## Background

SCAD is defined as a non-traumatic, non-atherosclerotic separation within the epicardial coronary artery wall leading to the development of a false lumen (FL) and eventually an intramural hematoma (IMH). Blood flow in the true lumen (TL) can be reduced by external compression, resulting in clinical manifestations [1]. CAS is a reversible vasoconstriction that narrows the lumen of normal or atherosclerotic coronary arteries, impairing myocardial blood flow.

Recent estimates suggest that SCAD accounts for between 2 and 4% of all ACS cases, predominantly affecting young women [1]. However, CAS was estimated at 50% in patients presenting with angina and 57% in those presenting with ACS [2].

To date, few reports have described an association between these two conditions [3, 4], and to the best of our knowledge, this is the first case with a comprehensive intravascular study (IVUS and OCT) demonstrating their simultaneous presence in the same vascular territory.

## Case presentation

A 36-year-old woman presented to the emergency department with acute, retrosternal pain. She reported similar episodes over the past month and had experienced a syncopal episode following a bereavement event. She had no coronary risk factors. She had given birth more than two years previously.

Electrocardiography and echocardiography were within normal limits. Laboratory tests showed an elevated troponin level (1576 ng/ml, n.v. <11). The patient was referred to the catheter laboratory with a diagnosis of non-ST elevation acute myocardial infarction. Aspirin and loading dose of ticagrelor were administered.

**Fig. 1:**
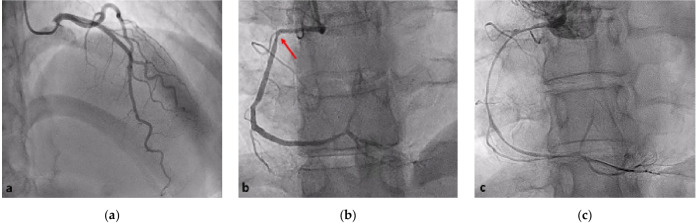
Index coronary angiography. **a** Left coronary angiogram shows no lesions; **b** the red arrow indicates discrete lesion of RCA resembling type 3 (focal stenosis) SCAD; **c** RCA diffuse spasm occurred immediately after wiring

**Fig. 2:**
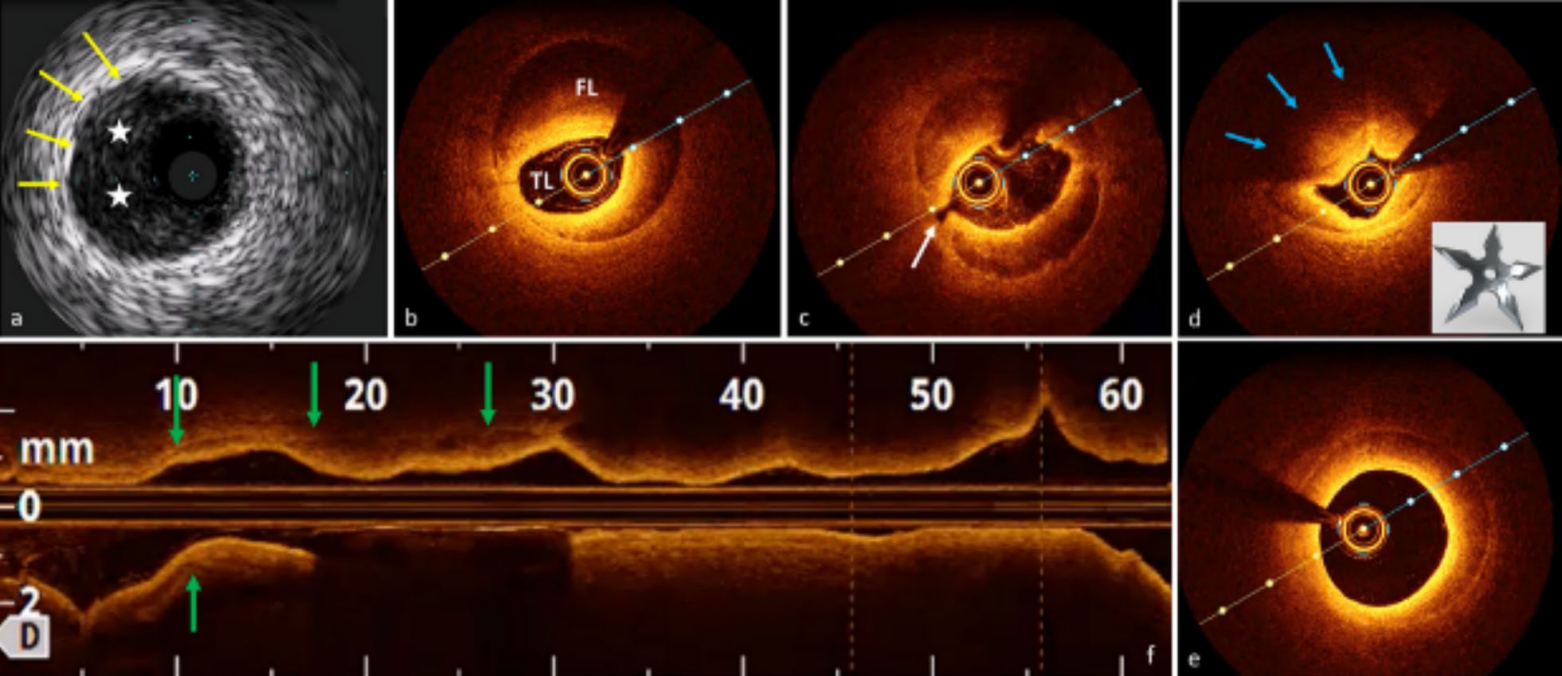
IVI of SCAD and CAS: **a** IVUS shows a crescent-shaped cloth (white stars). **b**–**e** OCT highlights the dissection (**b**) separating the TL from the FL; probable fenestration (**c**, white arrow); the “shuriken effect” of the TL compressed by IMH and CAS with reduced light penetration (light blue arrows) (**d**); the volume reduction of IMH at RCA para-ostium (**e**). Longitudinal view (**f**) showing IMH distribution

**Fig. 3:**
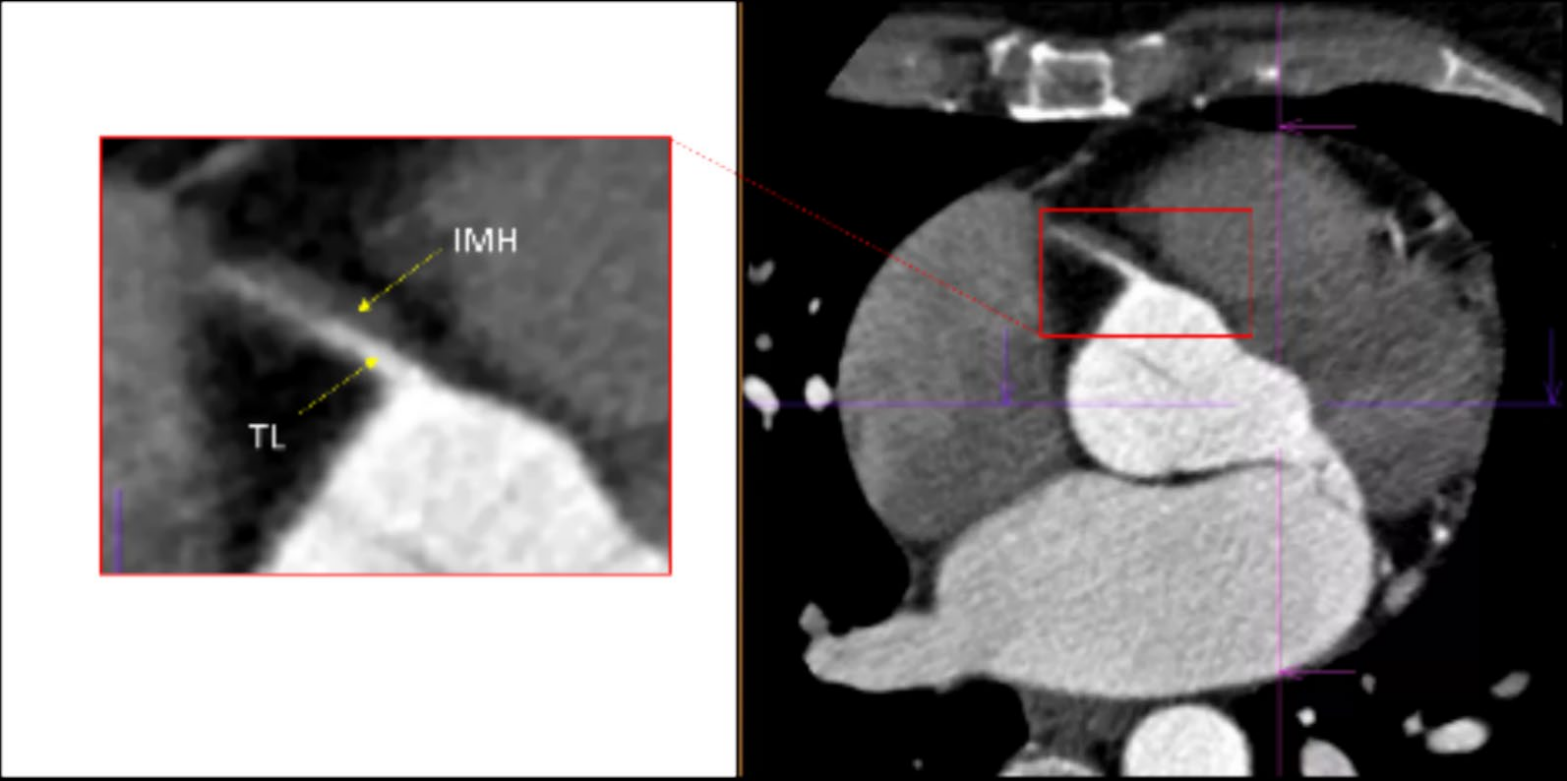
Cardiac computed tomography angiography. CCTA clearly depicts the TL and the IMH ruling out potential coronary occlusion and, above all, confirming RCA ostium sparing, assuring us about progression of the disease to the ascending aortas

**Fig. 4:**
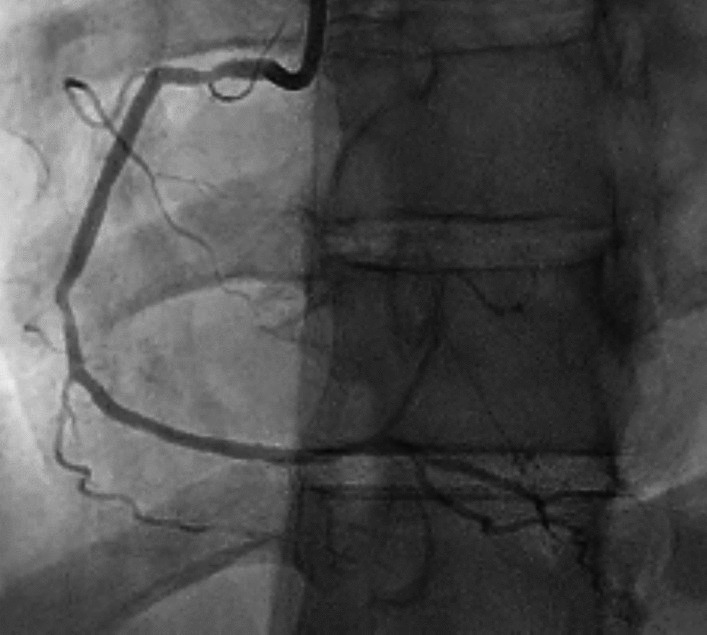
Pre-discharge CAG. RCA shows improved angiographic calibers

The left coronary angiogram showed unobstructed coronary arteries (Fig. 1a). The right coronary cannulation, however, drew more attention for a slight damping after ostium engagement and the subsequent angiogram showed a mild stenosis at the end of the first tract (Fig. 1b). Immediately after wiring with Runthrough ^TM^ NS Floppy, we observed extensive spasm unresponsive to intracoronary nitrates (Fig. 1c). IVUS showed a large amount of organized cloth in contact with the adventitia surrounding the probe and sparse echo-free spaces (Fig. 2a). OCT documented increased vessel diameters due to SCAD and IMH, which compressed the true lumen, spearing the ostium (Fig. 2b–f). We decided to adopt a supportive and watchful waiting strategy with prescription of acetylsalicylic acid, beta-blocker, and anxiolytics. Serial cardiac enzyme measurements showed gradually decreasing levels. On the fourth night of hospitalization, she had a sudden onset chest pain associated with ST segment elevation in the inferior leads. Intravenous nitrates were administered with prompt resolution of symptoms and ECG. The following day, CCTA excluded disease progression (Fig. 3). During the remaining stay, she was always asymptomatic and hemodynamically stable performing a two-week rehabilitation cycle. The patient was discharged with acetylsalicylic acid and beta-blocker on the twenty-third day of hospitalization, when angiography showed SCAD partial healing (Fig. 4). At six months of follow-up, she reported excellent quality of life and absence of chest pain.

## Conclusions

This case highlights the limits of angiography and the crucial role of combined IVI in the early diagnosis of SCAD (which can sometimes be masked by other conditions, such as CAS), the management of which significantly diverges from that of atherosclerotic ACS or vasospastic angina. It also highlights the importance of close inpatient monitoring due to the risk of disease extension and complications for which there are no clear predictors. Finally, CCTA may be the ideal surveillance tool for follow-up, especially in conservatively managed patients, as it is a noninvasive, low-risk study.

In our case, angiography showed nonspecific images that could have been ignored or mistaken for subcritical atherosclerotic lesions. IVUS immediately ruled out CAD and showed a large amount of organized IMH in contact with the adventitia, extending over almost the entire probe run (Fig. 2a). At this point, some doubts remained: Could we definitively rule out the possibility of intraluminal thrombus? Was the course of the guidewire completely in the TL? Furthermore, could we exclude the presence of intimal tears, especially near the RCA ostium? Subsequent imaging with OCT confirmed the diagnosis of SCAD and superimposed CAS: The classic crescentic semi-lunar FL compressed the TL in a star-shaped image that we called “Shuriken effect” (Fig. 2d, the famous ninja weapon). The entire intima-lumen interface was unaffected by thrombotic formations, showing only in the second tract some continuity solutions (the so-called “fenestrations,” Fig. 2c) connecting the FL with the TL. Finally, the OCT pullback clearly depicted a progressive reduction of the IMH volume until its complete disappearance about 5 mm from the RCA ostium (Fig. 2e), reassuring us of the possibility of SCAD extension into the ascending aorta.

Considering both the clinical status (patient was asymptomatic and hemodynamically stable) and anatomical parameters (peri-ostium sparing, thrombolysis in myocardial infarction flow 3 and absence of intraluminal thrombus), we decided to adopt a conservative approach with close monitoring. Currently, based on expert opinion, overall conservative treatment is the preferred management strategy in SCAD patients, unless revascularization is mandated (i.e., in the case of ongoing ischemia, hemodynamic instability, or left main coronary dissection) [1]. In fact, percutaneous coronary intervention is associated with high failure rates and poor outcomes in the setting of compromised arterial wall integrity [5–7]; furthermore, in most cases (70% to 90%) the natural history of SCAD appears to be spontaneous healing within 4 to 6 weeks [5], with subsequent architectural changes that may lead to late stent malapposition or graft failure [8].

Although the prognosis for long-term survival is favorable [1, 7], these patients are at high risk of chronic angina, coronary spasm, and SCAD recurrence [9]. In this scenario, the risk-benefit ratio must be carefully assessed before repeating CAG, and CCTA has been proposed as a valid noninvasive alternative to CAG. Although there is a possibility of missing distal small-vessel disease, CCTA may conversely be of greater benefit in patients with large vessel proximal SCAD, as in our case [1, 10]. Therefore, we opted for noninvasive delayed imaging with CCTA during the patient’s unique in-hospital anginal episode. This imaging methodic clearly depicted the dissection (Fig. 3), allowing us to definitively exclude the possibility of local disease extension, right coronary sinus involvement, or disease progression to other territories, and to identify CAS as the precipitating event.

Among the precipitating factors of ACS, SCAD is a unique entity that still has several “gray areas” in clinical practice. Conversely, CAS is a transient condition, often associated with SCAD, that may mimic some features of the latter, leading to a misleading diagnosis. Our findings suggest that combined IVI may help to clarify these scenarios, by providing unique and specific insights into the most relevant morphologic changes of the vessel, improving diagnostic accuracy and guiding the management strategy. Finally, CCTA represents a noninvasive and effective way to reassess SCAD lesions and to make a differential diagnosis with CAS, moreover in medically managed patients.

## Data Availability

Raw data for images are not publicly available to preserve individuals’ privacy under the European General Data Protection Regulation.

## Abbreviations

ACS: Acute coronary syndrome
CAS: Coronary artery spasm
CCTA: Cardiac computed tomography angiography
FL: False lumen
IMH: Intramural hematoma
IVI: Intravascular imaging
IVUS: Intravacular ultrasound
OCT: Optical coherence tomography
SCAD: Spontaneous coronary artery dissection
TL: True lumen
